# ﻿Complete mitochondrial genome of the abyssal coral *Abyssoprimnoagemina* Cairns, 2015 (Octocorallia, Primnoidae) from the Clarion-Clipperton Zone, Pacific Ocean

**DOI:** 10.3897/zookeys.1183.109000

**Published:** 2023-10-31

**Authors:** Romain Gastineau, Przemysław Dąbek, Kamila Mianowicz, Valcana Stoyanova, Artur Krawcewicz, Tomasz Abramowski

**Affiliations:** 1 Institute of Marine and Environmental Sciences, University of Szczecin, ul. Mickiewicza 16a, Szczecin, 70-383, Poland University of Szczecin Szczecin Poland; 2 Interoceanmetal Joint Organization, ul. Cyryla i Metodego 9-9A, Szczecin, 71-541, Poland Interoceanmetal Joint Organization Szczecin Poland

**Keywords:** Deep sea, environmental baseline studies, IOM, ISA, key species

## Abstract

The Clarion-Clipperton Zone (CCZ) in the tropical East Pacific is a region of interest for deep-sea mining due to its underwater deposits of polymetallic nodules containing economically important metals such as nickel, copper, and cobalt. It is also a region of extensive baseline studies aiming to describe the state of the environment, including the biodiversity of the benthic fauna. An abundant component of the abyssal plain ecosystem consists of sessile fauna which encrusts polymetallic nodules and are vulnerable to potential impacts arising from exploitation activities, particularly removal of substrate. Therefore, this fauna is often considered to have key species whose genetic connectivity should be studied to assess their ecological resilience. One such species is *Abyssoprimnoagemina* Cairns, 2015, a deep-sea coral from the CCZ whose presence in the Interoceanmetal Joint Organization (IOM) claim area has been confirmed during samplings. In this study, we used next-generation sequencing (NGS) to obtain the *18S* nuclear rRNA gene and the complete mitochondrial genome of *A.gemina* from IOM exploration area. The mitogenome is 18,825 bp long and encodes for 14 protein coding genes, 2 rRNAs, and a single tRNA. The two phylogeny reconstructions derived from these data confirm previous studies and display *A.gemina* within a highly supported cluster of seven species whose mitogenomes are all colinear and of comparable size. This study also demonstrates the suitability of NGS for DNA barcoding of the benthic megafauna of the CCZ, which could become part of the IOM protocol for the assessment of population diversity and genetic connectivity in its claim area.

## ﻿Introduction

The Clarion-Clipperton Zone (CCZ) is a large abyssal area beyond the national jurisdiction in the northern tropical part of the Pacific Ocean, west of Mexico and California and south-east of Hawaii, and governed by the International Seabed Authority (the ISA). This zone has been the area of scientific and industrial interest since the 1950s and is famous for the largest known deposits of polymetallic nodules—concretions containing various metals, including manganese, iron, copper, nickel, and cobalt (Van Nijen et al. 2018)—covering the seafloor (ISA 2010; Kuhn and Rühlemann 2021). It is also the largest region of exploration activities undertaken by ISA contractors and the scientific community and, as of now, the best recognized region for abyssal plain exploration in terms of environmental conditions (Amon et al. 2022).

Due to the growing interest in exploitation for nodules, an increasing number of sampling campaigns have been taking place in this area, all conducted under the legal framework of UNCLOS, the 1994 Agreement, and a set of Exploration Regulations of the ISA (in place since 2000; ISA 2015). Efforts are underway to develop regulations and establish a legal framework for deep-sea mining to ensure best environmental practices and to prevent, reduce, and control pollution and other hazards, which can potentially interfere with the ecological balance of the marine environment. Research expeditions are ongoing to study the biodiversity and ecological significance of the CCZ to better understand the potential impacts of mining activities and inform environmental management and conservation efforts.

The seafloor is inhabited by endemic species, with an increasing number being discovered and taxonomically described as exploration continues. A large majority of them represent invertebrates such as amphipods, annelids, or ophiuroids (e.g. Amon et al. 2016; Riehl and De Smet 2020; Bonifácio et al. 2021; Bribiesca-Contreras et al. 2021, 2022; Durden et al. 2021; Jones et al. 2021; Laming et al. 2021; Lejzerowicz et al. 2021; Neal et al. 2022; Stewart et al. 2023; Uhlenkott et al. 2023). The CCZ also hosts large benthic foraminifera, including sessile Xenophyophores attached to the nodules (Kamenskaya et al. 2012, 2015, 2017; Gooday et al. 2018, 2020, 2021; Goineau and Gooday 2019; Stachowska-Kamińska et al. 2022; Gooday and Wawrzyniak-Wydrowska 2023).

The CCZ seafloor is also home to an endemic species of deep-water coral, *Abyssoprimnoagemina* Cairns, 2015 (Cairns 2016), which lives attached to the nodules. The genus *Abyssoprimnoa* is for now known to be monospecific. A major morphological feature of this taxon is the presence of polyps that always occur in pairs, hence its species name *gemina* (Eng. “twins”). Its type locality lies within the German exploration area (BGR claim area), but specimens have also been documented in the neighbouring UK claim area (UKSRL I), all situated in the eastern part of the CCZ.

**Figure 1.**
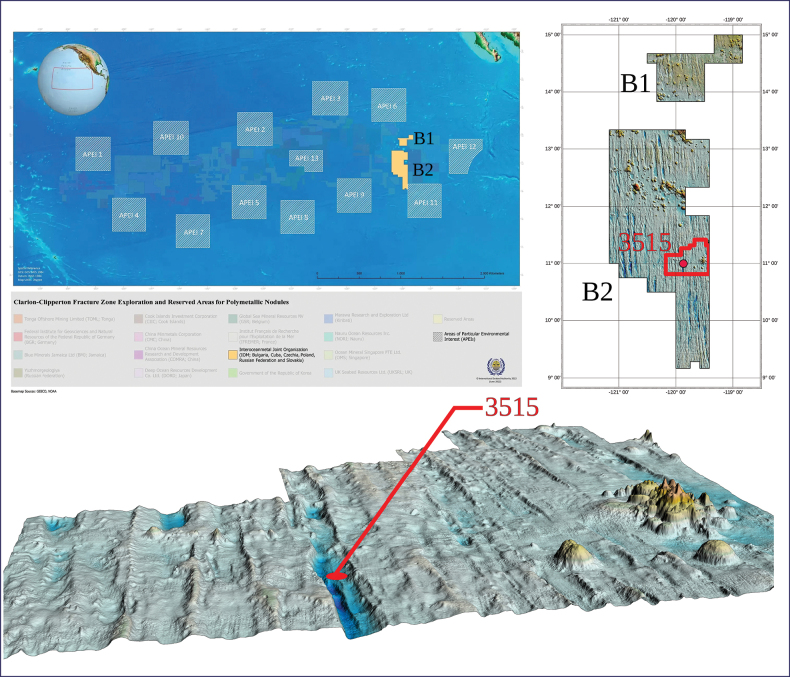
Upper left: location of the IOM claim area in the Clarion-Clipperton Zone, the Pacific Ocean (in orange) (https://www.isa.org.jm/maps/interoceanmetal-joint-organization/); upper right: bathymetric map of the IOM claim area B1 and B2, with the location of the exploration block H22 in the B2 claimed area (delimited by red lines) and the location of 3515 sampling station (red dot with the number); bottom: a block diagram of seafloor relief in the exploration block H22 (red dot – location of 3515 sampling station).

The eastern part of the CCZ is also where the Interoceanmetal Joint Organization exploration area (IOM claim area) is located (Fig. 1). The IOM claim area is ca 75,000 km^2^, at depths of 2,450–4,750 m, and has been explored by IOM since 1987 (Baláž 2022). Among the various topics of environmental baseline research (e.g. Skowronek et al. 2021; Hikov et al. 2022; Milakovska et al. 2022; Štyriaková et al. 2022), benthic megafauna and nodule-fauna studies are of crucial importance. The main sampling methods applied include photo profiling (video and still imagery documentation) and box-coring of the seafloor. Photo profiling documents the occurrence of benthic organisms living on and just above the seafloor.

**Figure 2.**
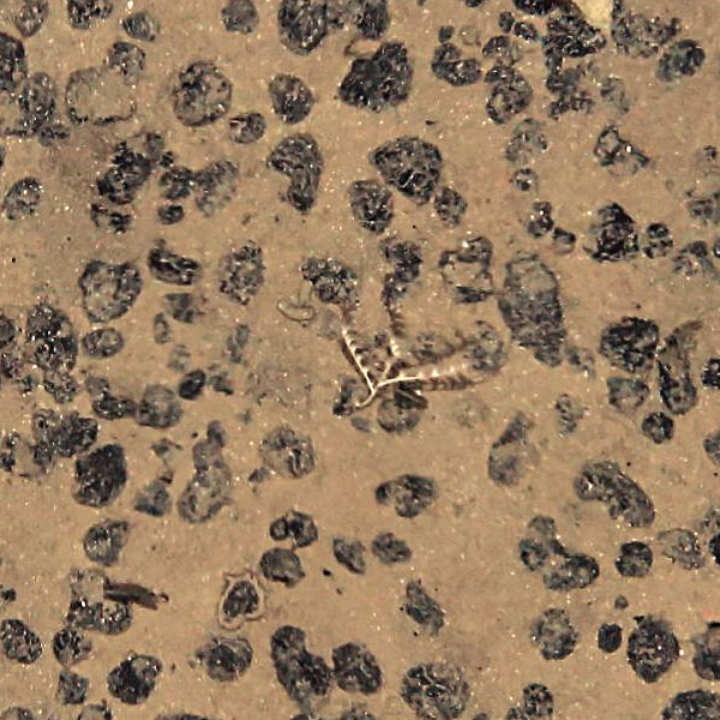
*Abyssoprimnoagemina* in-situ, as encountered in the IOM exploration zone at ca 4000 m depth (2014).

**Figures 3.**
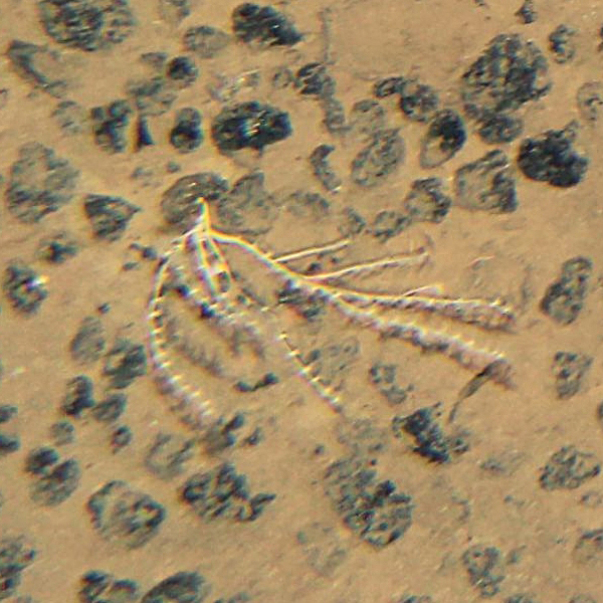
*Abyssoprimnoagemina* in-situ, as encountered in the IOM exploration zone at ca 4000 m depth (2014).

**Figure 4.**
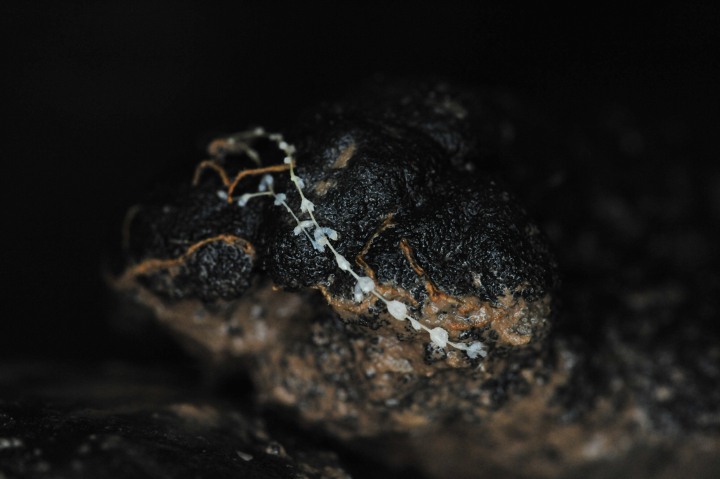
A specimen of *Abyssoprimnoagemina* collected from the CCZ on a nodule.

**Figure 5.**
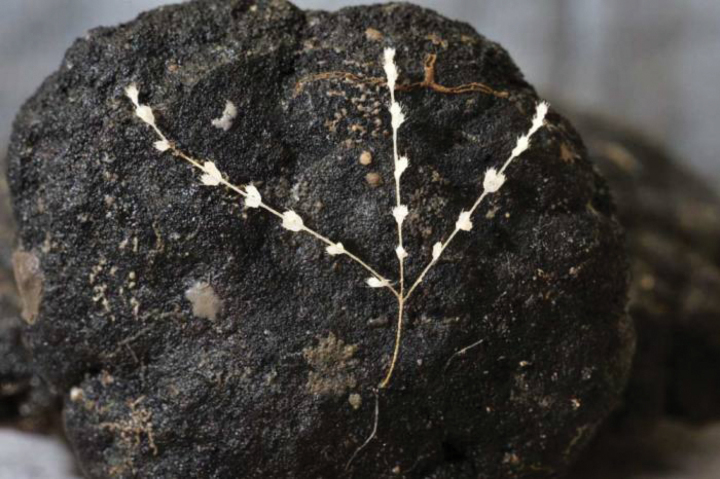
Same specimen of *Abyssoprimnoagemina* with outstretched branches.

Based on this method, the presence of *A.gemina* in the IOM claim area was suggested as early as in 2014 (Figs 2, 3) (IOM Megafauna Atlas, published online in 2021, https://iom.gov.pl/environmental-research/megafauna-atlas/) and confirmed later on in the same year by box-coring (Figs 4, 5), before its formal description by Cairns (2016).

During box-corer sampling, undisturbed seafloor sediment, and nodules were retrieved on board of the research vessel and all specimens of benthic mega-, macro-, and nodule fauna were sorted, photographed, registered, and then conserved in ethanol. Attempts were made to taxonomically identify each collected specimen on the basis of its morphology to the lowest level possible. This preliminary identification was planned to be complemented using molecular barcodes (one or more), such as the commonly used genes 18S (of the nuclear small subunit of the ribosomal RNA) or *cox 1* (the mitochondrial cytochrome c oxidase subunit 1 gene).

Although initially not mandatory (ISBA/19/LTC/8), DNA barcoding of specimens from the key taxonomic group (i.e. in the recommendations limited to the representatives of megafauna size class) is currently included in the 2019 “Recommendations for the guidance of contractors for the assessment of the possible environmental impacts arising from exploration for marine minerals in the Area” (ISBA/25/LTC/6/Rev.2 2022), which are considered obligatory for ISA contractors. Contractors are obliged to “assess regional distribution of species and communities/assemblages as well as genetic connectivity of key and representative species” (section III(b)(15)(d)). Furthermore, it is underlined that: “it is important that molecular studies be undertaken in conjunction with morphological taxonomic analyses” (Annex, 32(a)). However, no specific recommendation is given regarding which molecular marker should be used.

Following these recommendations, IOM recently developed and implemented a DNA-barcoding protocol, which was first applied to the samples obtained during the two exploration campaigns in 2014 and 2019. Principally based on the amplification and sequencing of the two genes mentioned above (*18S* and *cox1*) on all specimens except large Foraminifera, the protocol also tries to use, whenever possible, next-generation sequencing (NGS).

Prior to the current study, there were six sequences of *A.gemina* in GenBank, all of them being partial genes. These sequences originated from two studies. The first one exemplifies the use of DNA barcoding on the CCZ benthic fauna with a focus on Cnidaria (Dahlgren et al. 2016) and is based on two molecular markers, one nuclear (*18S*), and one mitochondrial (*16S*). The second study (Cairns and Wirshing 2018), which was aimed at a large scale multigene phylogeny of Primnoidae, compared with their classification based on morphological characters and relied on four markers, two nuclear (*18S* and *28S*) and two mitochondrial (*cox1* and *mutS*).

A specimen of *A.gemina* was sampled in the IOM area in 2014 and identified under a stereomicroscope a few years later based on the formal description by Cairns (2016). In the frame of the development of a DNA barcoding protocol for the IOM, it was subjected to NGS in 2021. Although it was uncertain if the analysis would be successful due to the time spent by the sample in ethanol, the complete mitogenome and *18S* gene of *A.gemina* were obtained.

The present article describes these findings and their comparison with existing references. The two phylogeny reconstructions derived from the mitogenome confirm the previous placement. The belonging of *A.gemina* to key species is introduced, and the interest of the intergenic regions of the mitogenome for population genetic studies on this species is discussed.

## ﻿Materials and methods

### ﻿Exploration and sampling

The specimen used in this study was sampled during the 2014 IOM cruise on 04/24/2014 at station 3515 (−119.7990, –11.1258), at 4241 m depth (Fig. 1). Specimens were acquired by a double shovel box-corer with a surface area of 0.25 m^2^ and penetration depth of 50 cm. The habitat type at the sampling station was described as sediments and polymetallic nodules (according to the ISA habitat type classification, ISBA/21/LTC/15/Corr.1 2021). The specimen was photographed, removed from the nodule it was attached to, moved to a 2.0 mL eppendorf tube with 96% ethanol for storage at 4 °C before being registered under the accession number 2014_28 in the benthic fauna collection of the IOM.

### ﻿Photography

Photographs of the sea bottom (Figs 2, 3) were taken using a Neptune TS-M1 towed on 8000 m long coaxial wireline. The camera set consists of pressure hull with the CANON EOS 60D camera inside (effective matrix resolution of 18 megapixel). The photo frame (automatic mode) was taken at pre-set time interval (20–30 s) when the current sonar data coincided with a given distance of 4 m above the sea floor, enabling to get photographs of the bottom surface with areal size of about 5 m^2^. Photographs of the specimen brought on board (Figs 4, 5) were taken with a Nikon D700 camera equipped with an AF-S MICRO Nikkor 105mm 1:2.8G ED lens.

### ﻿Sequencing and bioinformatic analyses

DNA was extracted using a DNeasy Blood & Tissue extraction kit from Qiagen from a 0.8 cm fragment of *A.gemina*. DNA was then sent to the Beijing Genomics Institute (BGI) in Shenzhen (China) where it was sequenced on a DNBSEQ platform. The 60 million 150-bp paired-end reads were assembled using SPAdes v. 3.15.5 (Bankevich et al. 2012) with a k-mer of 125. The mitochondrial genome and the *18S* gene were extracted from the contigs file by blastn command line analyses (Camacho et al. 2009). Annotation of the mitogenome was made with the help of MITOS (Bernt et al. 2013) with the genetic code 4 (mold, protozoan, and coelenterate mitochondrial code). The map of the mitogenome was generated using OGDRAW (Lohse et al. 2013).

### ﻿Phylogeny

Two phylogenetic analyses were conducted based on two recent works on octocorals (Muthye and Lavrov 2021; Muthye et al. 2022). The first one was based on the concatenated sequences of all the mitochondrial protein coding genes (PCG), and the second one on the amino-acid sequence of the DNA repair protein encoded by the *MutS* gene. Datasets from Muthye et al. (2022) were downloaded from OSF and appended with the corresponding sequences of *A.gemina*. All alignments were performed with MAFFT v. 7 (Katoh and Standley 2013) and the -auto option before being trimmed by trimAl v. 1.4.rev15 (Capella-Gutiérrez et al. 2009) and the -automated1 option. For the PCG, the genes were aligned separately, trimmed, and then concatenated using Phyutility v. 2.7.1 (Smith and Dunn 2008). The best model of evolution was chosen with ModelTest-NG (Darriba et al. 2020) on the protein alignment and the concatenated alignment. All maximum-likelihood phylogenies were conducted using IQ-TREE v. 2.2.0 (Minh et al. 2020) with 1000 ultra-fast bootstrap replications in each case.

### ﻿Data resources

The mitochondrial genome and the *18S* gene were submitted to GenBank with accession numbers OR197546 and OR192930, respectively. The raw fasta sequence of the mitogenome and *18S* gene, the annotated gbk file for the mitogenome, the alignments used for phylogeny and the complete trees in Newick format were deposited on Zenodo (https://doi.org/10.5281/zenodo.8100227).

## ﻿Results

### ﻿Sequencing, assembly, and phylogeny reconstructions

It was possible to retrieve the complete mitochondrial genome with redundant endings from the contigs file. However, for unknown reasons, the operon of nuclear rRNA appeared in several pieces that failed to merge. Thus, only the complete *18S* gene (1,833 bp) was extracted from the contigs file and later deposited in GenBank (Accession number: OR192930).

**Figure 6.**
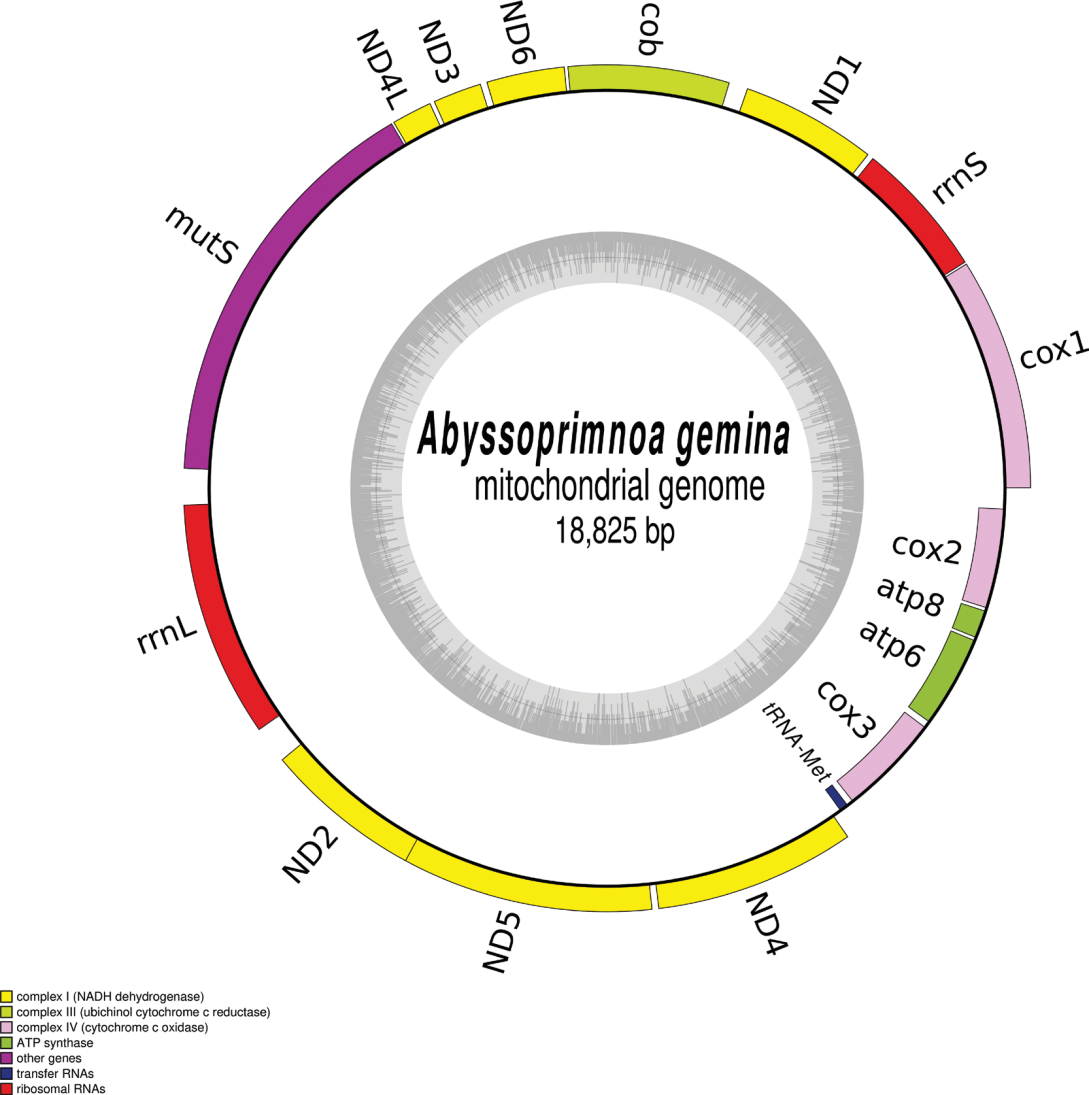
Map of the mitochondrial genome of *Abyssoprimnoagemina.* The type of genes (e.g. complex I–IV genes, rRNA genes) is represented by different colours whose meaning is explained on the bottom left part. The grey circle represents the GC content.

**Figure 7.**
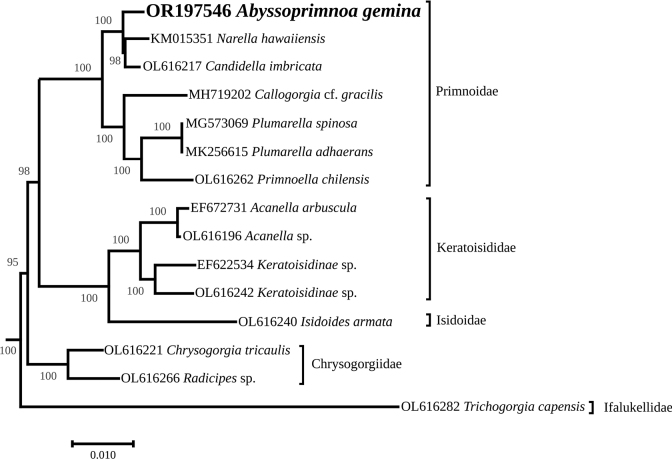
Maximum-likelihood phylogenetic tree obtained from an alignment of concatenated mitochondrial protein-coding genes of 185 taxa of octocorals. The subtree corresponds to a highly supported clade containing among other the family Primnoidae. The name of the families is indicated next to the brackets. The bootstrap values are indicated at the nodes, and the GenBank accession number of the sequence is indicated before the species name. The scale represents the number of substitutions per site.

**Figure 8.**
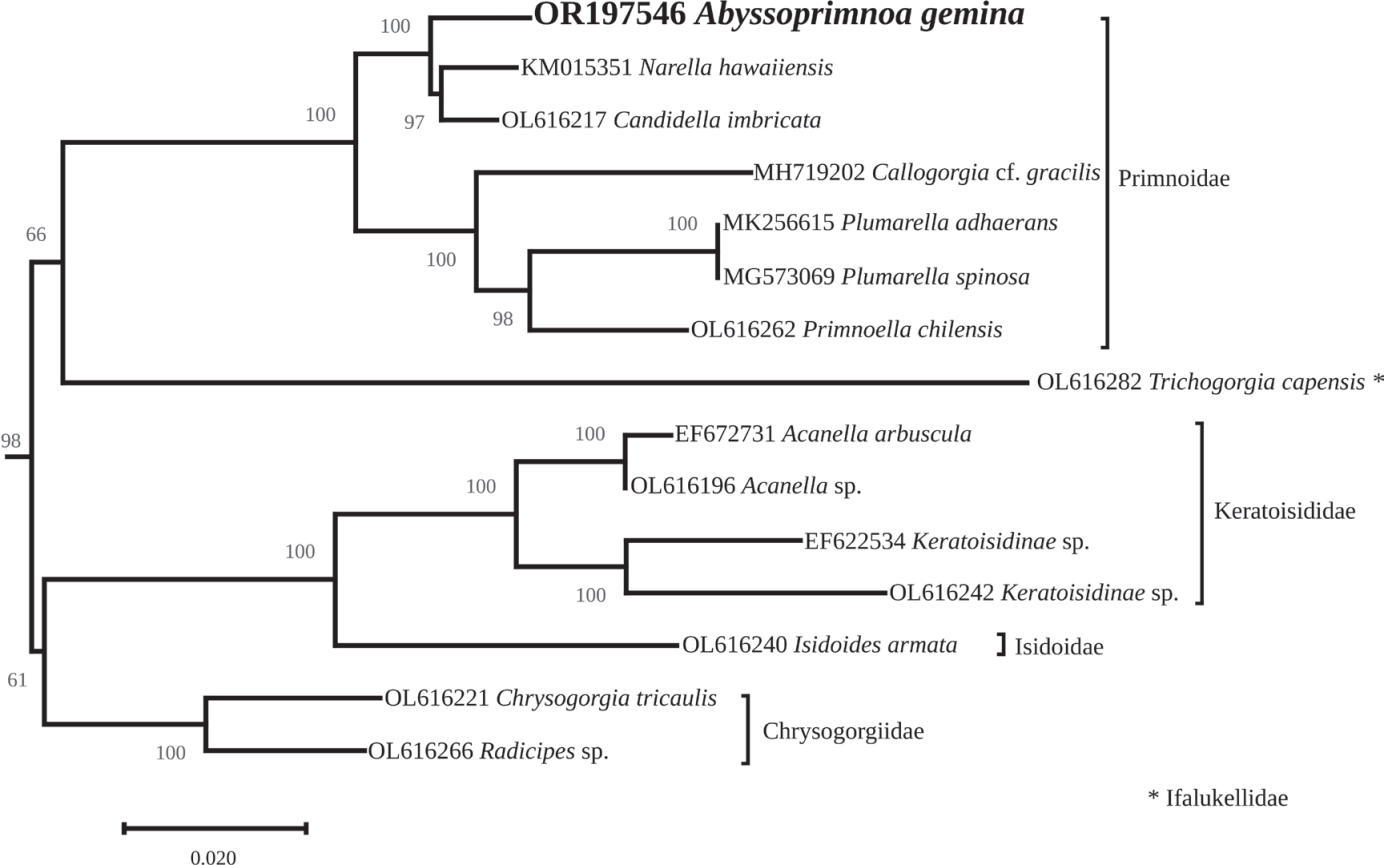
Maximum-likelihood phylogenetic tree obtained from an alignment of 183 *MutS* encoded protein. The subtree corresponds to the highly supported clade containing among other the family Primnoidae. The name of the families is indicated next to the brackets. The bootstrap values are indicated at the nodes, and the GenBank accession number of the sequence is indicated before the species name. The scale represents the number of substitutions per site.

The complete mitogenome is 18,825-bp long (GenBank: OR197546). It encodes for 14 protein coding genes, 2 rRNAs, and a single tRNA (Fig. 6), which is congruent with other octocorals. The best models of evolution returned by ModelTest-NG were TVM+I+G4 for the concatenated PCG and HIVW+I+G4+F for the *MutS* encoded protein. The complete trees, which were too large for publication, are available as indicated in the data resources statement. The figures represent instead highly supported subtrees containing five families. *Abyssoprimnoagemina* is displayed in both phylogeny reconstructions in a highly supported clade (100%) with all the six other species of the Primnoidae family (Figs 7, 8). It is noteworthy that the mitogenome of *A.gemina* is also colinear with these Primnoidae. There are discrepancies in the rest of the subtrees for what concerns the position of the family Ifalukellidae, represented here by *Trichogorgiacapensis* (Hickson, 1904) (Hickson 1904). It is also noteworthy that the support at the nodes for the MutS-inferred tree is in general lower when compared to the multigene tree. It especially concerns two nodes, one associating Primnoidae and Ifalukellidae (66%), and the other associating Chrysogorgiidae to Keratoisididae + Isidoidae.

### ﻿Comparison with available sequences: DNA barcoding

Comparison of the sequences of the mitochondrial PCG with the references from GenBank showed a complete identity. The sequences concerned were the partial *cox1* (MG986971) and *MutS* (MG986922) genes. For what regards the partial mitochondrial 16S gene (KX384626), there were two polymorphisms found at the nine last base pairs of the 3' ending. There was a single indel in the 5' ending of sequence KX384618, differentiating it from the 18S complete gene obtained in the course of this study. Both sequences, however, differed from MG980108 by the presence of a 9-bp deletion at the 3' ending of the sequence.

## ﻿Discussion

As stated above, the mitogenomes of *A.gemina* and the other six Primnoidae species are colinear, clustering together in both phylogeny reconstructions. The size of the *A.gemina* mitogenome (18,825 bp) is slightly smaller compared to the other ones, which range between 18,838 bp for *Narellahawaiiensis* Cairns & Bayer, 2008 (Cairns and Bayer 2008; Figueroa and Baco 2015) to 19,037 bp for *Plumarellaspinosa* Kinoshita, 1907 (Knioshita 1907; Choi et al. 2020).

To our knowledge, *A.gemina* is so far only the second representative of the CCZ benthic fauna to have its mitochondrial genome sequenced. This can be easily accounted for by the scarcity and small sample sizes, as well as the difficulty to accurately identify them taxonomically. Due to the small size of most benthic macrofauna specimens, obtaining a sufficiently large amount of DNA might result in the destruction of large fragments of their colonies, which may be a problem when depositing voucher specimens. In addition, in the case of the IOM samples, it must be remembered that they had been kept in ethanol for seven years prior to DNA extraction, which could have jeopardized the outcome of the sequencing by lowering the quantity and quality of DNA to be used as a template for the preparation of the library.

During this study, we noticed that an NGS-based approach could be considered when the size of the specimen is large enough and belongs to a known species, even if the biological material is not fresh. In addition to the original aim of performing DNA barcoding on the CCZ samples for comparison with databases, we also obtained the complete mitogenome and obtained phylogeny reconstructions, which provide more evidence for the proximity between *A.gemina* and the genus *Narella* Gray, 1870, as already demonstrated by Cairns and Wirshing (2018).

As mentioned above, *A.gemina* was not the first organism from the CCZ benthos to be studied using NGS, as five years earlier the mitochondrial genome of the demosponge *Plenastercraigi* Lim & Wiklund, 2017 (Lim et al. 2017) had been obtained by Taboada et al. (2018a). Contrary to our study, which is based on a single individual, the subsequent article from Taboada et al. (2018b) was based on 75 Porifera specimens. Their aim was to find nuclear microsatellite loci among the contigs obtained after assembly for studies at the population level, and to test their distribution among other specimens by PCR.

Taboada et al. (2018b) justified their approach on the grounds of the small numbers of polymorphic sites in the mitochondrial genome of Porifera. A similar problem is likely to occur in octocorals, such as with *A.gemina*. The high conservation level of octocoral mitogenomes has already been noted by several authors (Muthye and Lavrov 2021; Muthye et al. 2022; Ramos et al. 2023), which can be a problem in population genetics. There is an absolute conservation observed among mitochondrial PCG of our sample and others in the databases. Even if polymophisms were spotted between the rRNA genes, they were always located at the endings of the reference sequences, all obtained by Sanger sequencing. Although not rejecting the possibility that these polymorphisms do exist, more samples need to be sequenced to confirm this.

An additional interest of sequencing a complete mitogenome is that it also includes the non-coding parts. As stated above, the conservation of mitochondrial genes among Anthozoa is reputedly high, leading authors to deem them as less efficient for studies at the population level than the nuclear gene (Shearer et al. 2002). This is exemplified by *Balanophylliaelegans* Verrill, 1864 (Verrill 1864) for which partial *cox1* gene has been found invariable among 18 populations distributed along 3,000 km (Hellberg 2006). However, studies on mitochondrial intergenic regions were used to define haplotypes among Anthozoa (Smith et al. 2004). For example, up to eight haplotypes were found for *Galaxeafascicularis* (Linnaeus, 1767) (Watanabe et al. 2005). Smith et al. (2004) studied a non-coding region between *cox1* and *cox2*, while Watanabe et al. (2005) used the intergenic spacer between *cob* (or *cytb*, coding for cytochrome b) and *ND2* (coding for the second subunit of the NADH dehydrogenase). In *A.gemina*, there is also a 155 bp intergenic spacer between *cox1* and *cox2* (both genes located on different strands), but *cob* and *ND2* are not contiguous in this species. Instead, we found other intergenic sequences of potential interest, for example between *ND1* - *cob* (131 bp) and *rrnL* – *ND2* (265 bp). Although shorter than the regions sequenced by the aforementioned authors, they could still present polymorphisms of interest.

In addition, reads such as those obtained in the course of this study can also be processed through pipelines such as HybPiper (Johnson et al. 2016) supplemented with a proper database (e.g. Quek et al. 2020) to directly extract from them orthologous loci putatively polymorphic. The data gathered can be later employed in phylogenomic studies as recently exemplified by Quek et al. (2023), but they might also be used for studies at the population level.

During the exploration phase of the CCZ, the contractors are obliged to gather environmental data (describing the state of environment) to inform the baseline against which possible environmental impacts arising from exploration for marine minerals will be assessed (ISBA/25/LTC/6/Rev.2). Collecting information on “regional distribution of species and communities/assemblages as well as genetic connectivity of key and representative species” is one of the requirements imposed on the contractors by the regulator; however, there is no recommendation with respect to the criteria for identifying key species. Rabone et al. (2023) proposed a list of key taxonomic groups of macrofauna (tanaids, isopods, and polychaetes), as well as megafauna (asteroids and ophiuroids). The term “key” as used here means most abundant and dominant (“major”) groups of benthic fauna (Simon-Lledó et al. 2020). To our knowledge, there is limited information on connectivity of benthic fauna in the CCZ, whether it refers to key species or not (Taboada et al. 2018b; Kersten et al. 2019; Janssen et al. 2019; Patel et al. 2020; Bribiesca-Contreras et al. 2021). It represents one of the knowledge gaps presently identified (Amon et al. 2022), which must be addressed in order to inform evidence-based environmental management. The point of the current study also belongs to IOM’s strategy to deal with this issue in the eastern part of the CCZ.

If we refer to existing works, *P.craigi*, the aforementioned species of demosponge found on polymetallic nodules, was proposed by Taboada et al. (2018a, 2018b) as a key species and a model organism to study biogeographic patterns at varying spatial scales, with the help of molecular and genomic tools. The three reasons behind this choice (Taboada et al. 2018b) were: (1) a high dependence on polymetallic nodules which provide the substrate for adults, (2) a filter-feeding mechanism of obtaining nutrients from particles suspended in water, and thus high vulnerability to increased turbidity, also resulting from collector plumes (Weaver et al. 2022), and (3) presumed (analogous to other sponges) short pelagic larval durations (lecithotrophic larvae) with relatively limited dispersal ability. Combination of these three traits of *P.craigi* can, in turn, result in high vulnerability of this species to temporal (increased turbidity) and permanent (substrate removal) changes in environmental conditions caused by deep-sea mining.

*Abyssoprimnoagemina* shares at least two (or three if lecithotrophic larvae are confirmed) of these traits (in general coral larvae are considered lecithotrophic (Rodd et al. 2022)), and therefore we propose that it can be considered a key species. In general, cold-water corals, including deep-sea corals, are regarded vulnerable to anthropogenic impacts (e.g. Cordes et al. 2016). In addition to the traits already mentioned, these corals are also characterized by slow growth and recovery rates (Roark et al. 2009).

If *A.gemina* is to be later considered a key species, it will imply that further genetic investigations will have to be conducted on it, building on the current work. Depending on the results of the next IOM cruises, perhaps additional specimens of *A.gemina* will become available for sequencing. The other ISA contractors and members of the scientific community working on the CCZ could also participate to improve our knowledge on *A.gemina* from their own exploration area following the protocol described here.

## ﻿Additional information

### Author ORCIDs

Romain Gastineau https://orcid.org/0000-0001-8661-5118

Przemysław Dąbek https://orcid.org/0000-0002-3736-3011

Kamila Mianowicz https://orcid.org/0000-0002-7755-3258

Tomasz Abramowski https://orcid.org/0000-0002-9029-406X

### Data availability

All of the data that support the findings of this study are available in the main text.
